# Edible native plants of the Gulf of Mexico Province

**DOI:** 10.3897/BDJ.10.e80565

**Published:** 2022-06-03

**Authors:** Eva María Piedra-Malagón, Victoria Sosa, Diego F. Angulo, Milton H. Díaz-Toribio

**Affiliations:** 1 Jardín Botánico Francisco Javier Clavijero, Instituto de Ecología A.C., Xalapa, Veracruz, Mexico Jardín Botánico Francisco Javier Clavijero, Instituto de Ecología A.C. Xalapa, Veracruz Mexico; 2 Red de Biología Evolutiva, Instituto de Ecología A.C., Xalapa, Veracruz, Mexico Red de Biología Evolutiva, Instituto de Ecología A.C. Xalapa, Veracruz Mexico

**Keywords:** ethnobiology, edible plants, seasoning, packaging, ethnic groups, Tabasco, Tamaulipas, Veracruz

## Abstract

**Background:**

Currently, at the global level, human food is mainly based on a few crops extensively cultivated as monocultures. Climate change, changes in land to agriculture and cattle raising, as well as the scarcity of water all affect and reduce the possibility of cultivating alternative crops. One way to face this global problem is to promote the knowledge, production and consumption of native food species on a regional scale.

For this study, two databases were constructed for the Gulf of Mexico Province: 1) edible plant species with their corresponding common name, category of use, plant organ(s) utilised as food and type of management; 2) distribution records of these edible species. These species, in addition to being part of the biological diversity of Mexico are of high nutritional, cultural and gastronomical value and have been present in the diet of the inhabitants and ethnic groups in the region since pre-Hispanic times.

**New information:**

This study presents the native edible plants of the Gulf of Mexico Province, an area inhabited by 15 ethnic groups. The main novelty of this contribution is the edible plant species database, which includes the records of 482 species that belong to 101 families and 268 genera. We also present information rarely reported in an ethnobotanical inventory: 1) category of food use, 2) category of plant organ used, 3) common name, 4) type of management and 5) the georeferenced distribution of species occurrence in the Gulf of Mexico Province.

## Introduction

It is estimated that, of the 374,000 plant species recorded in the planet (Christenhusz and Byng 2016), 30,000 are edible (Garn and Leonard 1989, Corlett 2016). Of these edible plants, only 7,000 have been either cultivated or collected in their original habitats (Collins and Hawtin 1999, Chivenge et al. 2015). Surprisingly, over 60% of man's diet is based mainly on 20 species which meet 90% of the world's food requirements (Bacchetta et al. 2016). There is much interesting documentation of under-utilised edible plants in several regions around the world to promote their cultivation and collection and, more importantly, to emphasise their importance in food security and nutrition (Kang et al. 2013, Sánchez-Mata and Tardío 2016, Bhatia et al. 2018, CEPAL 2018, Ozturk et al. 2018, FAO 2019, Dop et al. 2020, Jacob et al. 2020, Ulian et al. 2020).

Over 15% of the plants consumed as food in the world originated in Mexico (Saruhkán et al. 2009), where of the 7,461 vascular plant species recorded as useful, 2,168 are edible (Mapes and Basurto 2016). The vast majority of these edible plants are wild species, which, in addition to being part of the biological diversity, are also a fundamental part of the culture and history of each of the country’s regions. The richness of wild edible plants is reflected in the great and diverse traditional Mexican cuisine, which UNESCO has listed as Intangible Cultural Heritage of Humanity (Silva et al. 2016, Iturriaga 2018).

The biocultural diversity that characterises Mexico is clearly represented across the territory, particularly in the biogeographical province of the Gulf of Mexico where the diverse communities of 15 ethnic groups (Ayapanecos, Chinantecos, Choles, Chontales, Huastecos, Mazatecos, Nahuas, Olutecos, Otomies, Sayultecos, Tepehuas, Tzeltales, Texistepequeños, Totonacas and Zoques) inhabit in an area of 17,350 km^2^, from the State of Tamaulipas in the north to the States of Veracruz and Tabasco in the south (Atlas de los Pueblos Indigenas de México 2020). This Province is characterised by coastal regions on the Gulf of Mexico, diverse ecosystems and elevations that rise from the coastal plains to tall mountains. The native people of this Province have inherited and maintained the use and knowledge of wild edible plants to this day (Hernández et al. 1991, Del Angel-Pérez and Mendoza 2004, Ruíz-Carrera et al. 2004, Lascurain et al. 2010, Sánchez-Trinidad 2017, Centurión-Hidalgo et al. 2019).

Many studies on the native edible plants of Mexico have focused on the Maya of the Yucatan Peninsula (Fedick et al. 2008, Cáceres and Cruz 2019), the Nahua from Central Mexico (Casas et al. 1996, Gutiérrez-­Santillán et al. 2019) and the ethnic groups of desert regions, such as the Tepehuanos and the Yaquis (Narváez-Elizondo et al. 2020, Ramírez García et al. 2020). The focus of this paper is to record the native edible plants used in food preparation and to wrap food in the Province of the Gulf of Mexico, for which studies are scarce. Two databases were compiled based on fieldwork, data from the main herbaria of the region and from the ethnobotanical literature. A checklist of edible species, their location in this Province, based on a georeferenced database, their common names, the type of use and the plant parts utilised are also included.

## General description

### Purpose

This contribution provides information on the native plants that are used in food preparation in the Gulf of Mexico Province. These species, in addition to representing a biological resource, are of great historical and cultural value in the gastronomy of 15 ethnic groups and the rural and urban inhabitants of the Gulf of Mexico region. The data are expected to increase awareness about the immense potential that wild edible plants offer as a food resource and to promote the conservation of the species that grow in areas under strong anthropogenic pressure.

## Project description

### Study area description

According to biogeographic studies, the Province of the Gulf of Mexico, located in the Neotropical Region, extends as a continuous strip along the coastal plain of the Gulf of Mexico, covering the States of Veracruz and Tabasco and the southern part of the State of Tamaulipas that borders the Tropic of Cancer (Morrone 2005, Morrone 2010). Small areas of the States San Luis Potosí, Hidalgo, Puebla, Oaxaca, Chiapas and Campeche, as well as northern Belize and Guatemala, are also part of the Gulf of Mexico Province (Morrone 2005). As most of the territory of this Province lies in Tamaulipas, Veracruz and Tabasco, our study focuses on this area. The climate in the Gulf of Mexico Province is variable and ranges from tropical warm (in the lowlands and coastal areas) and temperate (in the central lands), to cold (in the highlands and mountainous areas), with an average annual temperature of 25ºC and an average annual rainfall of 1,500 mm (Soto et al. 2001, Soto-Esparza and Giddings-Berger 2011). The coastal strip of the Gulf of Mexico Province increases in orographic complexity towards the interior of the continent, with its highest elevations reaching around 2,000 m in the El Cielo Biosphere Reserve in Tamaulipas (Steinberg et al. 2014) and 3,500 m a.s.l. in Veracruz, close to the Cofre de Perote and Pico de Orizaba volcanoes, which have elevations of 4,282 and 5,747 m a.s.l., respectively (Jáuregui and Soto 1975). The elevation gradient of the Gulf of Mexico Province has allowed six types of vegetation to establish: coniferous forest, xerophilic scrub, cloud forest, deciduous tropical forest, evergreen tropical forest and aquatic and underwater vegetation (Challenger and Soberón 2008). The Gulf of Mexico Province is one of the regions with the greatest diversity of vascular plants with 5000 species in Veracruz (Victoria Sosa, com. pers), 4667 species in Tamaulipas (Arturo Mora-Olivo, com. pers) and 3500 in Tabasco (Carlos M. Burelo, com. pers). This region is considered one of the best areas for agricultural activities (Córdoba y Ordóñez 2004). The intensive cultivation of corn, sorghum, cotton, rice, sugar cane, coffee, banana, citrus fruits, pineapples and livestock are the main economic activities of the region (Travieso-Bello et al. 2006). However, the high impact of land use change has drastically altered and reduced ecosystems to mostly degraded environments and secondary forests (Mendoza-González et al. 2012, Steinberg et al. 2014).

## Sampling methods

### Sampling description

Data were obtained from fieldwork carried out for a project on the edible plants of the Francisco Javier Clavijero Botanical Garden, by consulting the specimen database of the XAL Herbarium of the Instituto de Ecología A.C. and the biodiversity database of the National Biodiversity Commission (CONABIO), as well as an exhaustive literature search of ethnobotanical, agronomic, ethnographical and floristic studies. With these data, we compiled a checklist of the food plant species for the three States within the Gulf of Mexico Province: Tabasco, Tamaulipas and Veracruz. In the supplementary files (Suppl. material 3), the 125 bibliographical sources consulted here, published from 1979 to 2020, are included.

For this study, we only recorded species native to the Gulf of Mexico Province; introduced plants were not considered. Based on Roullier et al. (2013) who indicated the evolution of populations of *Ipomoeabatatas*, we decided to include this species. The majority of the plant species utilised for food are native and often collected in the wild, while some other species, such as those of the central region of the Gulf of Mexico, in Veracruz, are locally cultivated on a small scale or in home gardens to facilitate their accessibility to rural families and ethnic groups (Chablé-Pascual et al. 2015, Cruz and López 2018, Rooduijn et al. 2018, López-Santiago et al. 2019, Aguilar Vásquez et al. 2019, Avilez-López et al. 2020).

**Category of use and plant organ utilised as food**. Several categories have been proposed to identify the use of plants and represent artificial divisions originating from an ethnocentric perspective. Here, the following are used: edible, spicy, medicinal, utilised in religious ceremonies and ornamental (Bye and Linares 1983). In this study, plants used as food are divided into four categories:

1. species eaten raw or after some preparation process, such as cooking, food colourants, drinks, etc.,
2. species added to food as seasoning due to their pleasant taste or aromatic qualities or flavouring properties,
3. species used as packaging to wrap or contain food (solid and/or liquid) for cooking, transportation and/or tasting and
4. a multipurpose category assigned to species reported in more than one category (edible, seasoning and packaging).

For instance, *Piperauritum*, which is known as “acuyo” or “hoja santa”, is considered a multipurpose species because the leaves are utilised as flavouring in soups and for wrapping up certain meals. *Zeamays*, “maíz,” is another example of a multipurpose species; its fruits and seeds are eaten and its leaves are dried and used to transport food and keep it fresh and its dried leaves are also used to wrap the dough for “tamales”.

Nine edible plant organs were identified: 1) roots or bulbs, 2) stem, 3) bark, 4) wood or trunk, 5) leaves, 6) flowers, 7) fruits, 8) seeds, 9) resin/latex and 10) multipurpose, a category assigned to species for which more than one organ is utilised as food. For instance, in *Sechiumedule*, different organs are eaten and prepared in different ways. Its growing tendrils are called “guías de chayote” and cooked in sauces, its rhizome is known as “chayotextle” and mainly eaten fried and the fruits are known as “chayote” or “christophine” and eaten as cooked vegetables. For certain beans (*Phaseoluscoccineus*, *P.vulgaris*) and squashes or gourds (*Cucurbitaargyrosperma*, *Cucurbitapepo*), the fruits, seeds and flowers are cooked and eaten.

**Type of management**. The categories proposed by Bye (1993) and Caballero et al. (1998) were followed:

1. gathered directly from their ecosystems,
2. incipient management in which plants are tolerated or even promoted and
3. cultivated locally in orchards or home gardens.

## Geographic coverage

### Description

**Figure 1.**
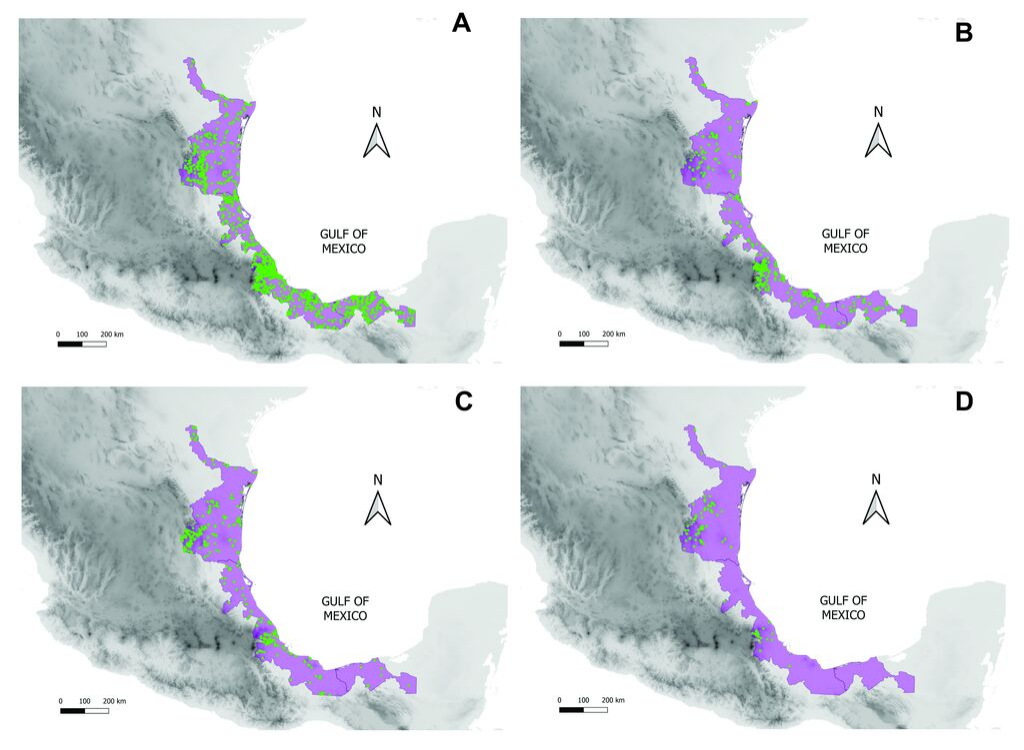
Map showing the location of the Gulf of Mexico Province and the distribution of the occurrence records of edible native species of the four most diverse families. The map is based on herbarium and GBIF data. **A** Leguminosae; **B** Solanaceae; **C** Cactaceae; **D** Asparagaceae.

The Province of the Gulf of Mexico is located in the Neotropical Region, it extends from sea level to 3,500 m above sea level and is a continuous strip along the coastal plain of the Gulf of Mexico from the States of Tabasco and Veracruz in the south to the southern portion of the State of Tamaulipas in the north of Mexico (Fig. 1).

### Coordinates

17.143085 and 27.682536 Latitude; -100.142119 and -17.256033 Longitude.

## Taxonomic coverage

### Description

The classification of families and orders follows APG IV (2016). The species names were reviewed, based on the World Flora Online (Borsch et al. 2020) and for those species that are not on this list, the name is cited as given in the corresponding reference or herbarium specimen. All the specimens collected in the field were identified to the species level and their vouchers deposited in the XAL Herbarium, curated by the Instituto de Ecología, A.C. in Xalapa, Mexico. The geographic coordinates of native edible plants of the Gulf of Mexico Province (Suppl. material 2) were obtained from GBIF.org (2021) using the gbif function in the dismo package for R (Hijmans et al. 2017, R Core Team 2020). We acknowledge there is some variation regarding coordinate precision, even though the records downloaded from GBIF were either original or interpreted, we only considered the records taken from herbarium specimens. Many herbarium specimens were collected in the past centuries; therefore, they did not include coordinate systems. However, these historical records that did not include precise georeferences were considered when they were added precisely, because they indicated the precise locality.

## Usage licence

### Usage licence

Creative Commons Public Domain Waiver (CC-Zero)

## Data resources

### Data package title

Data package of Edible native plants of the Gulf of Mexico Province.

### Number of data sets

2

### Data set 1.

#### Data set name

Categories of use, plant organ utilised and management of native edible plants of the Gulf of Mexico.

#### Description

Database with information about categories of use, plant organ utilised and categories of management of native edible plants of the Gulf of Mexico Province. Available as Suppl. material 1.

**Data set 1.** 

Column label	Column description
Family	Scientific name of the family in which the taxon is classified.
Species	Full scientific name of the taxon.
Category of use (Edibles)	use = 1, not used = 0.
Category of use (Seasonings)	use = 1, not used = 0.
Category of use (Packaging)	use = 1, not used = 0.
Category of use (Multipurpose by category of use)	use = 1, not used = 0.
Plant organ utilised (Root or bulb)	use = 1, not used = 0.
Plant organ utilised (Stem)	use = 1, not used = 0.
Plant organ utilised (Bark)	use = 1, not used = 0.
Plant organ utilised (Wood or trunk)	use = 1, not used = 0.
Plant organ utilised (Leaf)	use = 1, not used = 0.
Plant organ utilised (Flower)	use = 1, not used = 0.
Plant organ utilised (Fruits)	use = 1, not used = 0.
Plant organ utilised (Seed)	use = 1, not used = 0.
Plant organ utilised (Resin or latex)	use = 1, not used = 0.
Plant organ utilised (Multipurpose by plant organ used)	use = 1, not used = 0.
No data of plant organ used	Out data = 1, with data = 0.
Management	category or categories of management.

### Data set 2.

#### Data set name

Geographical coordinates of edible native plants of the Gulf of Mexico Province

#### Description

Geographical coordinates of edible native plants distributed in the Gulf of Mexico Province. Available as Suppl. material 2.

**Data set 2.** 

Column label	Column description
Family	The full scientific name of the family in which the taxon is classified.
Genus	The full scientific name of the genus in which the taxon is classified.
Species	The scientific name of the specie.
Decimal Lat	Geographical coordinates in decimal (latitude).
Decimal Lon	Geographical coordinates in decimal (longitude).
gbifID	Reference number of gbif database.

## Additional information

### Results

**Figure 2.**
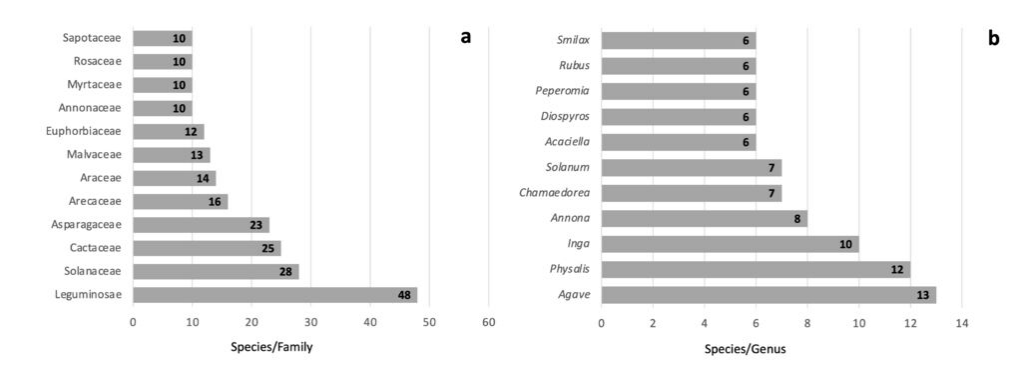
Diversity of the native food species of the Gulf of Mexico Province, classified by family (**a**) and genus (**b**).

**Edible species by group.** We recorded a total of 482 of plants species used as food in the Gulf of Mexico Province and belonging to 101 families and 268 genera (Table 1). The families with the highest number of native edible species were Leguminosae (48 species), Solanaceae (28 species), Cactaceae (25 species), Asparagaceae (23 species), Arecaceae (16 species), Araceae (14 species), Malvaceae (13 species), Euphorbiaceae (12 species) and Annonaceae, Myrtaceae, Rosaceae and Sapotaceae (10 species) (Fig. 2a). These 12 families comprise 219 species, representing 45% of the native edible plants recorded for the Gulf of Mexico Province. The other 55% are distributed unevenly amongst the remaining 88 families. The most important genera were *Agave* (13 species), *Physalis* (12 species), *Inga* (10 species), *Annona* (8 species), *Chamaedorea* and *Solanum* (7 species), *Acaciella*, *Diospyros, Peperomia, Rubus* and *Smilax* (6 species) (Fig. 2b). These 11 genera include 87 species, representing 18% of the native edible plants recorded for the Gulf of Mexico. The other 82% are distributed unevenly amongst the remaining 257 genera.

**Figure 3.**
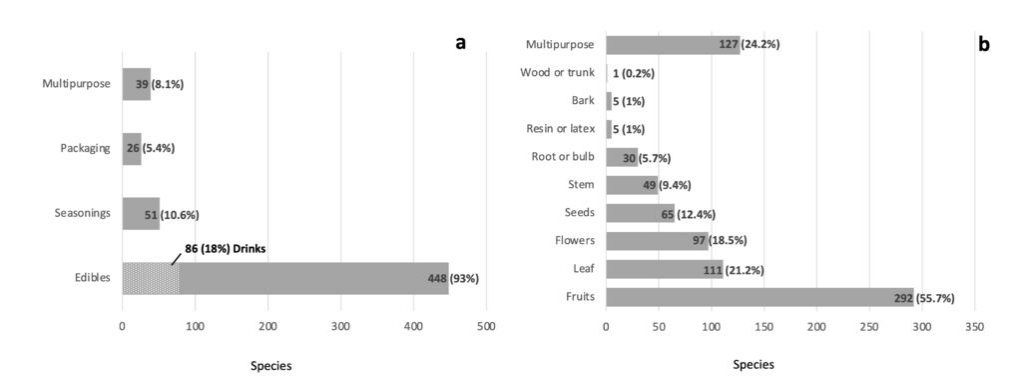
Categories of use (**a**) and plant part used (**b**) for the native edible plants of the Gulf of Mexico Province.

**Categories of native plant use**. Edible species were the majority with 448 species (93%), which includes the 86 species (18%) used in the production of drinks, 51 species as seasonings (10%) and 26 species (5%) as packaging. The remaining 39 species (8%) are utilised in more than one category (Fig. 3a, Suppl. material 1).

**Edible plant organs**. Fruits were the organs most commonly used as food with 292 species (55.7%), followed by leaves with 111 species (21%), flowers with 97 species (18.5%), seeds with 65 species (12.4%), stems with 49 species (9.4%) and roots and bulbs with 30 species (5.7%). Resin or latex, bark and wood had five, five and one species, respectively. For 127 species (24%), more than two organs were utilised as food, seasoning or packaging (Fig. 3b, Suppl. material 1).

**Figure 4.**
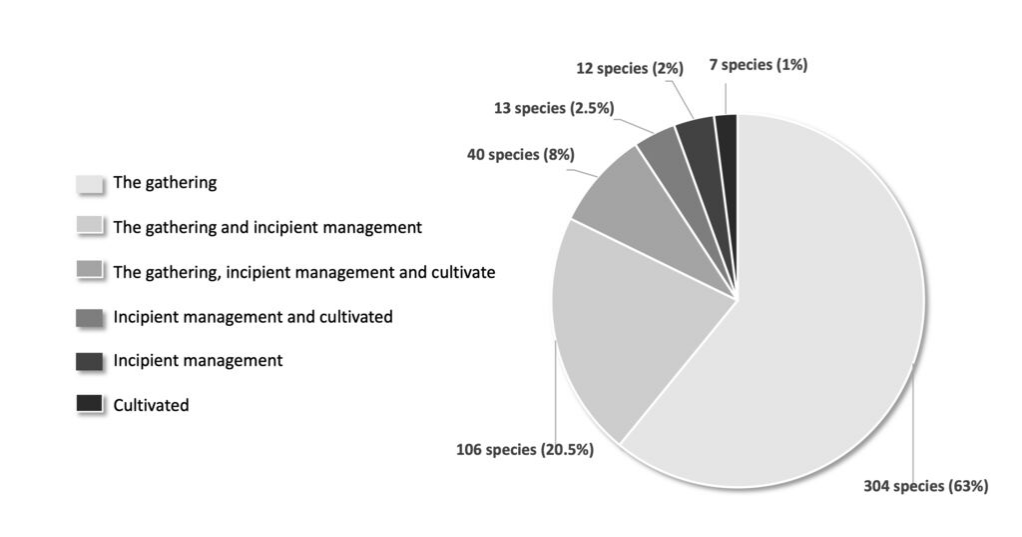
Management categories for the native edible plants of the Gulf of Mexico Province.

**Management categories**. Most of the species are gathered in the wild (304 species, 59%), with fewer species in the categories of incipient management (12 species, 2.3%) and cultivated (7 species, 1.3%). Interestingly, we found that a notable number of species (159, 33%) are under a combination of two or three different management strategies in the study area (Fig. 4, Suppl. material 1).

## Supplementary Material

85B0835E-C871-5520-BC11-EABC38362710 10.3897/BDJ.10.e80565.suppl1 Supplementary material 1 Categories of use, plant organ utilised and management of native edible plants of the Gulf of Mexico
Data type binary data Brief description Categories of use (edibles, seasonings, packaging and multipurpose by category of use), plant organ utilised (root or bulb, stem, bark, wood or trunk, leaf, flowers, fruits, seed, resin or latex and multipurpose by plant organ utilised) and categories of management of native edible plants of the Gulf of Mexico Province. The information are encoded as presence = 1 and absence = 0. File: oo_654412.csv https://binary.pensoft.net/file/654412 Eva María Piedra-Malagón, Victoria Sosa, Diego F. Angulo, Milton Díaz-Toribio

996A49E2-B714-52B6-BEC0-52A6633133A3 10.3897/BDJ.10.e80565.suppl2 Supplementary material 2 Geographical coordinates Data type occurrences Brief description Geographical coordinates of edible native plants in the Gulf of Mexico Province. File: oo_654413.csv https://binary.pensoft.net/file/654413 Eva María Piedra-Malagón, Victoria Sosa, Diego F. Angulo, Milton Díaz-Toribio

307406B3-1CB2-50D4-BDF8-836590F1ACFA 10.3897/BDJ.10.e80565.suppl3 Supplementary material 3 Bibliographical sources Data type References Brief description List of bibliographical references used to build the database of native edible plants of Gulf of Mexico Province. File: oo_633856.txt https://binary.pensoft.net/file/633856 Eva María Piedra-Malagón, Victoria Sosa, Diego F. Angulo, Milton Díaz-Toribio

## Figures and Tables

**Table 1.** Table 1. Alphabetic arrangement by family and species and common names of native edible plants of the Gulf of Mexico Province. The families correspond to those proposed by APG IV (2016).

Famillia	Scientific name	Common name
Acanthaceae	*Justiciaspicigera* Schltdl.	añil de piedra, flor de azulillo, hierba púrpura, hierba tinta, muicle, sangre de cristo
Actinidaceae	*Saurauiacana* B.T.Keller & Breedlove	ixtlahuatl, moco blanco, moquillo, pipicho
Adoxaceae	*Sambucuscanadensis* L.	sauco, saúco, sauco colorado, sauco extranjero, sauco rojo, xómetl
Amaranthaceae	*Amaranthuscruentus* L.	quelite blanco, tsaw juki (Totonaco)
Amaranthaceae	*Amaranthushybridus* L.	quelite, quelite morado, quintonil
Amaranthaceae	*Amaranthushypochondriacus* L.	quelite quintonil, tsaw (Totonaco)
Amaranthaceae	*Amaranthuspalmeri* S.Watson	quelite blanco, quintonil
Amaranthaceae	*Atriplexcanescens* (Pursh) Nutt	quelite blanco, quintonil
Amaranthaceae	*Chenopodiumincisum* Poir.	epazote morado
Amaranthaceae	*Chenopodiumberlandieri* Moq.	huazontle
Amaranthaceae	*Dysphaniaambrosioides* (L.) Mosyakin & Clemants	epazote, Ihkgna (Totonaco)
Amarylidaceae	*Alliumglandulosum* Link & Otto.	cebolla de monte, cebolleja
Amarylidaceae	*Alliumlongifolium* (Kunth) Spreng.	cebollín, cebollina blanca, chunacate
Anacardiaceae	*Pistaciamexicana* Kunth	lentisco
Anacardiaceae	*Rhusaromatica* Aiton	agrito
Anacardiaceae	*Rhusmicrophylla* Engelm.	agrito, correosa
Anacardiaceae	*Rhuspachyrrhachis* Hemsl.	agrito
Anacardiaceae	*Rhusvirens* Lindh. ex A.Gray	lantrisco
Anacardiaceae	*Spondiasmombin* L.	ciruela, jamsinpichcuy (Núntaha’yi), jobo, xípa (Totonaco)
Anacardiaceae	*Spondiaspurpurea* L.	ciruela criolla, ciruela roja, ciruelo, skgatán, tuxpana
Anacardiaceae	*Tapiriramexicana* Marchand	bienvenido, cacao, caobilla, jobo, nompi
Annonaceae	*Annonaglabra* L.	anona, anona blanca, anona de corcho
Annonaceae	*Annonaglobiflora* Schltdl.	anona del monte, chirimolla, chirimoyito, anonasilvestre, ashiwitmusni (Totonaco), guanabanilla
Annonaceae	*Annonamuricata* L.	guanábana, guanábano
Annonaceae	*Annonapurpurea* Moc. & Sessé ex Dunal	cabeza de negro, ilama, ilana, chincua, chincuya, soncuaya, zapote ilama
Annonaceae	*Annonareticulata* L.	akxitkiwi (Totonaco), anona, anona colorada, anona morada, anona roja, anonillo, anono, chirimoya, corazón de buey, ilama, morada, yati (Núntaha’yi)
Annonaceae	*Annonarensoniana* Standl.	ND
Annonaceae	*Annonascleroderma* Saff.	chirimuya
Annonaceae	*Annonasquamosa* L.	anona blanca, surumuya
Annonaceae	*Mosannonadepressa* (Baill.) Chatrou	nazareno prieto
Annonaceae	*Rolliniamucosa* (Jacq.) Baill.	chirimoya, akxitkiwi (Totonaco), chirimoyo, anona, anona montaña
Apiaceae	*Daucusmontanus* Humb. & Bonpl. ex Spreng.	culantrillo
Apiaceae	*Eryngiumfoetidum* L.	cilantro cimarrón, cilantro de espina, cilantroespinudo, extranjero, huitzcolanto, perejil, perejil criollo, strankgeyu (Totonaco)
Apocynaceae	*Gonolobusbarbatus* Kunth	tlayote
Apocynaceae	*Gonolobusniger* (Cav.) R.Br. ex Schult.	cahuayote, chompipe, chupipe, chupipi, papullo, talayote, uyk
Apocynaceae	*Marsdeniacoulteri* Hemsl.	talayote
Apocynaceae	*Marsdeniamacrophylla* (Humb. & Bonpl.) Fourn.	talayote
Apocynaceae	*Plumeriarubra* L.	cacalosúchil, cacalote, cacaloxochitl, floramarilla, flor de corpus, flor de cuervo, flor de mayo, flor de la Santa Cruz, flor de templo, gagaloxuchit, jacalos chil, kakaloxochitl, pu’uchmooya, súchel, súchil, xanath spiritu
Apocynaceae	*Tabernaemontanaalba* Mill.	cojón de gato
Apocynaceae	*Tabernaemontanalitoralis* Kunth	ND
Apocynaceae	*Vallesiaglabra* (Cav.) Link	mahuira
Araceae	*Anthuriumschlechtendalii* Kunth	panizbatl, tisbatl
Araceae	*Anthuriumscandens* (Aubl.) Engl.	ND
Araceae	*Monsteradeliciosa* Liebm.	piñanona
Araceae	Monsteratuberculatavar.tuberculata Lundell	ND
Araceae	Philodendronradiatumvar.radiatum Schott	ND
Araceae	*Philodendronsagittifolium* Liebm.	ND
Araceae	*Philodendrontripartitum* (Jacq.) Schott	ND
Araceae	*Pistiastratiotes* L.	ND
Araceae	*Spathiphyllumfriedrichsthalii* Schott	flor chile de gato, flor de gato, flor de San Lorenzo, oloxochitl, súchil, tolancaxochitl
Araceae	*Spathiphyllumphryniifolium* Schott	flor de chile, flor de chilillo, cuchijec
Araceae	*Syngoniumangustatum* Schott, Oesterr.	ND
Araceae	*Syngoniumneglectum* Schott	ND
Araceae	*Syngoniumpodophyllum* Schott	ND
Araceae	*Xanthosomarobustum* Schott	lokg, malvarón, oreja de elefante, rejalgar
Araliaceae	*Dendropanaxarboreus* (L.) Decne & Planch.	palo de agua, palo santo
Araliaceae	*Hydrocotyleumbellata* L.	pesetilla
Araliaceae	*Oreopanaxcapitatus* (Jacq.) Decne & Planch.	caballero, cabellera de palo, coamatl, choco, tablilla
Araliaceae	*Oreopanaxechinops* (Schldtl. & Cham.) Decne. & Planch.	caballero, cinco hojas, macuilillo, pata de gallo, siete hojas
Araliaceae	*Oreopanaxflaccidus* Marchal	choco, hoja de queso
Arecaceae	*Acrocomiaaculeata* (Jacq.) Lodd. ex Mart.	cocoyol, coyol, coyol redondo, guacoyul
Arecaceae	*Astrocaryummexicanum* Liebm. ex Mart.	chapay, chapaya, chichón, chipi, chocho, chocón, flor de chocho
Arecaceae	*Attalearostrata* Oerst.	corozo, coyol real, coyolito real, palma real
Arecaceae	*Bactrismajor* Jacq.	coyolillo, guiscoyol, jahuacté, jahuactillo
Arecaceae	*Bactrismexicana* Mart.	chiquiyul, jahuacté
Arecaceae	*Braheadulcis* (Kunth) Mart.	palma de abanico, palma de sombrero, palmilla, palmito
Arecaceae	*Chamaedoreaalternans* H.Wendl.	guaya de cerro, pampi, tepejilote
Arecaceae	*Chamaedoreacataractarum* Mart.	guaya de río, guayita de río
Arecaceae	*Chamaedoreaelegans* Mart.	jilote de cerro, tepejilote, tepexilotl
Arecaceae	*Chamaedoreaoblongata* Mart.	litámpa (Totonaco), tepejilote
Arecaceae	*Chamaedoreapinnatifrons* (Jacq.) Oerst.	guaya de cerro, pacaya
Arecaceae	*Chamaedoreaseifrizii* Burret	palmita bambu
Arecaceae	*Chamaedoreatepejilote* Liebm.	chive, jilote de cerro, joma, guaya, tepejilote, tepexilotl
Arecaceae	*Cryosophilastauracantha* (Heynh.) R.Evans	guano coba
Arecaceae	Reinhardtiagracilisvar.gracilior (Burret) H.E.Moore	coquillo
Arecaceae	*Sabalmexicana* Mart.	aptas, guano, palma, palma apachite, palmareal, palma redonda, palmito
Asparagaceae	*Agaveamericana* L.	maguey, maguey cenizo
Asparagaceae	*Agavefourcroydes* Lem.	ND
Asparagaceae	*Agavegentryi* B.Ullrich	agave, bayusa, cacaya, flor dejiote, flor de gigante, flor de henequén, flor de maguey, flor de mezcal, flor de pitol, flor de sotol, golumbos, gualumbos, hualumbos, huexote, kakaya, machete, maguey, quiote
Asparagaceae	*Agavelechuguilla* Torr.	maguey verde
Asparagaceae	*Agaveatrovirens* Karw. ex Salm-Dyck	lechuguilla
Asparagaceae	*Agavemapisaga* Trel.	maguey manso
Asparagaceae	*Agavemitis* Mart.	ND
Asparagaceae	*Agavemontana* Villarreal	maguey chino
Asparagaceae	*Agavemontium*-*sancticaroli* García-Mend.	jarcia
Asparagaceae	*Agavesalmiana* Otto ex Salm-Dyck	maguey manso
Asparagaceae	*Agavestriata* Zucc.	espadín
Asparagaceae	*Agaveweberi* Cels ex Poisson	maguey verde
Asparagaceae	*Beschorneriaseptentrionalis* García-Mend.	ND
Asparagaceae	Beschorneriayuccoidessubsp.dekosteriana (K.Koch) García-Mend.	gasparito
Asparagaceae	*Dasylirionlongissimum* Lem.	aracuete, padillo, vara
Asparagaceae	*Dasyliriontexanum* Scheele	sotol, sotol chino
Asparagaceae	*Manfredascabra* (Ortega) McVaugh	maguey, maguey cenizo
Asparagaceae	*Millabiflora* Cav.	azúcar de campo, azucena del campo, azucena, silvestre, estrella, flor de mayo, flor de San Juan, flor de San Nicolas, jacinto de monte
Asparagaceae	*Yuccacarnerosana* (Trel.) McKelvey	palma barreta, palma loca, palma samandoca
Asparagaceae	*Yuccafilifera* Chabaud	palma, palma barreta, palma china, palma samandoca, pita, pita amarilla
Asparagaceae	*Yuccagigantea* Lem.	akgalukut, cardum, chochas, chocho, flor de palma, flor de palmo, iksoxochitl, izote, izotl, izxote, kardum, palma, palmito, palmito guaya, yuca
Asparagaceae	*Yuccapericulosa* Baker	izote, palmito
Asparagaceae	*Yuccatreculeana* Carriere	palma, palma de d tiles, palma pita, pita verde
Asteraceae	*Dahliaimperialis* Roezl ex Ortgies	dalia
Asteraceae	*Helianthusannuus* L.	girasol, maíz de texas
Asteraceae	*Porophyllumruderale* (Jacq.) Cass.	papaloquelite, tepehua, venadilla
Asteraceae	*Senecioroseus* Sch.Bip.	lechuguilla
Asteraceae	*Tageteserecta* L.	cempasúchil, cempaxúchil, cempoal, cempoalxóchitl, flor de muerto
Asteraceae	*Tageteslucida* Cav.	hierbanis
Asteraceae	*Tagetesmicrantha* Cav.	anisillo
Asteraceae	*Tamaulipaazurea* (DC.) R.M.King & H.Rob.	limpia tuna
Asteraceae	*Thymophyllapentachaeta* (DC.) Small	limoncillo, parraleña
Bassellaceae	*Anrederavesicaria* (Lam.) C.F.Gaertn.	sacasil
Bataceae	*Batismaritima* L.	saladilla
Begoniaceae	*Begoniagracilis* Kunth	ND
Begoniaceae	*Begoniaheracleifolia* Schltdl. & Cham.	xocoyole, xkutn (Totonaco)
Begoniaceae	*Begoniamultistaminea* Burt-Utley	chucuyul, chucuyule
Begoniaceae	*Begonianelumbonifolia* Schltdl. & Cham.	begonia, quelite agrio, xocoyole
Berberidaceae	*Berberistrifoliolata* Moric.	palo amarillo
Bignoniaceae	*Amphitecnatuxtlensis* A.H.Gentry	ND
Bignoniaceae	*Crescentiaalata* Kunth	guaje, jícaro
Bignoniaceae	*Crescentiacujete* L.	guaje, tecomate
Bignoniaceae	*Parmentieraaculeata* (Kunth) Seem.	cuajilote, chote, chayote, guachilote, guajilote, pepino silvestre
Bignoniaceae	*Tecomastans* Kunth	San pedro
Bixaceae	*Bixaorellana* L.	achiote, axiote
Boraginaceae	*Cordiaalliodora* (Ruiz & Pav.) Oken	aguardientillo, bajón, baria, hormiguillo,hormiguero, huitl, sochicahua, sochíchi, solecillo, solerillo, suchil, tepesuchi, xulaxuchilt
Boraginaceae	*Cordiaboissieri* A.DC.	nacahua
Boraginaceae	*Cordiadentata* Poir.	baboso, gravel, gulaber, moquillo, olavere, zazamil
Boraginaceae	*Cordiadodecandra* A.DC.	cópite, trompillo
Boraginaceae	*Ehretiaanacua* (Teran & Berl) I.M.Johns	anácua, manzanilla
Boraginaceae	*Ehretiatinifolia* L.	beec, capulín cimarrón, frutillo, manzana, manzanilla, manzano, nandimbo, palo verde, roble, sauco, pingüico
Boraginaceae	*Tournefortiahirsutissima* L.	nigua, tlachichinole
Brassicaceae	*Lepidiumcostaricense* Thell.	lentejilla
Brassicaceae	*Lesquerellafendleri* (A.Gray) S.Watson	lesquerella
Bromeliaceae	*Aechmeamagdalenae* (André) André ex Baker	pita
Bromeliaceae	*Bromeliakaratas* L.	chichipo, chiyol, guapilla, piñuela
Bromeliaceae	*Bromeliapinguin* L.	borregos, borreguitos, cardo, cardón, guapilla
Bromeliaceae	*Catopsisnutans* (Sw.) Griseb.	gallitos
Bromeliaceae	*Greigiavan*-*hyningii* L.B.Sm.	piña cimarrona, piña de monte
Bromeliaceae	*Hechtiaglomerata* Zucc.	guapilla china
Burseraceae	*Burserasimaruba* (L.) Sarg.	chaca
Burseraceae	*Protiumcopal* (Schltdl. & Cham.) Engl.	copalillo, shewin'shan (Totonaco)
Cactaceae	*Acanthocereustetragonus* (L.) Hummelinck	cruceta, jacube, jacubo
Cactaceae	*Cylindropuntiaimbricata* (Haw.) F.M.Knuth	cardón
Cactaceae	*Echinocactusplatyacanthus* Link & Otto.	biznaga burra
Cactaceae	Echinocereuscinerascensvar.tulensis (Bravo) N.P.Taylor	alicoche San Juanero, pitaya San Juanera
Cactaceae	*Echinocereusenneacanthus* Engelm.	alicoche, biznaga
Cactaceae	*Echinocereuspentalophus* (DC.) Ruempler	alicoche
Cactaceae	*Echinocereusstramineus* (Engelm.) F.Seitz	alicoche, alicoche verde, pitahaia deagosto, pitahaya, pitahaya de agosto, pitaya, sanjuanera
Cactaceae	*Ferocactushamatacanthus* (Weber) Britton & Rose	biznaga de tuna
Cactaceae	*Ferocactuspilosus* (Salm-Dyck) Werderman	biznaga, biznaga roja
Cactaceae	*Hylocereusundatus* (Haw.) Britton & Rose	pitahaya, pitajaya, pitaya, pitaya orejona
Cactaceae	*Mammillariahemisphaerica* Engelm.	pichilingos
Cactaceae	*Mammillariaheyderi* Muehlenpf.	biznaga de chilitos
Cactaceae	*Marginatocereusmarginatus* (DC.) Backeb.	órgano
Cactaceae	*Myrtillocactusgeometrizans* (Mart. ex Pfeiff.) Console	garambullo
Cactaceae	*Neomammillariacandida* (Scheidw.) Britton & Rose.	biznagita blanca
Cactaceae	*Nopaleadejecta* Salm-Dyck	nopal, nopal chamacuero
Cactaceae	*Opuntiacantabrigiensis* Lynch	arrastradillo
Cactaceae	*Opuntiaengelmannii* Salm-Dyck ex Engelm.	nopal cuijo
Cactaceae	*Opuntiaficus*-*indica* (L.) Mill.	tuna, nopal blanco
Cactaceae	*Opuntialeucotricha* DC.	nopal duraznillo, duraznillo
Cactaceae	*Opuntiastenopetala* Engelm	arrastradilla
Cactaceae	*Pereskiopsisaquosa* (Weber) Britton & Rose	nopal de la punzada
Cactaceae	*Rhipsalisbaccifera* (Miller) Stearn	ND
Cactaceae	*Selenicereustestudo* (Karw. ex Zucc.) Buxb.	cruzeta
Cactaceae	*Stenocereusgriseus* (Haw.) Buxb.	pitayo
Calophyllaceae	*Mammeaamericana* L.	akgchixitjak (Totonaco), mamey amarillo, mamey santo domingo, zapote cabello, zapote domingo
Campanulaceae	*Lobeliaxalapensis* Kunth	berro silvestre, hierba loca
Cannabaceae	*Aphananthemonoica* (Hemsl.) J.-F.Leroy	chilesmin, cuachichile, cuerillo, pipín, quebrache, tomatillo, varilla
Cannabaceae	*Celtiscaudata* Planch.	carboncillo
Cannabaceae	*Celtisiguanaea* (Jacq.) Sarg.	cuerétaro, granjeno, tontu, uña de gato
Cannabaceae	*Celtislaevigata* Willd.	palo blanco
Cannabaceae	*Celtispallida* Torr.	granjeno
Cannabaceae	*Tremamicrantha* (L.) Blume	togalapoli
Cannaceae	*Cannaindica* L.	plantanilla
Capparidaceae	*Morisoniaamericana* L.	bandera, chachalaca, chilalaga, chimalaga, papatla, platanillo, papata
Caricaceae	*Caricapapaya* L.	papaya
Caricaceae	*Jacaratiadolichaula* (Donn.Sm.) Woodson	palo de agua, palo de pan, papaya cimarrona
Caricaceae	*Jacaratiamexicana* A.DC.	bonete, coalsuayote, cuaguayote, papaya demontaña, papaya orejona
Caricaceae	*Vasconcelleacauliflora* (Jacq.) A.DC.	melocotón, papayita, papaya cimarrona, papaya de monte, papaya oreja de mico
Caryophyllaceae	*Drymariacordata* (L.) Willd. ex Schult.	berro cimarrón, lengua de pájaro
Cecropiaceae	*Cecropiaobtusifolia* Bertol.	hormiguillo
Celastraceae	*Hippocrateavolubilis* L.	tecolote
Celastraceae	*Mygindalatifolia* Sw.	ND
Celastraceae	*Salaciacordata* (Miers) Mennega	gogo, tegualala
Celastraceae	*Salaciaimpressifolia* (Miers) A.C.Sm	bejuco zapote, tenguale
Celastraceae	*Wimmeriaconcolor* Cham. & Schltdl.	cuyuxquihui
Chrysobalanaceae	*Chrysobalanusicaco* L.	caimito, caco, ciruela de paloma, icaco, icaco de playa, jicaco, uva de mar
Chrysobalanaceae	*Couepiapolyandra* (Kunth) Rose	gurupillo, olo sapo, olozapote, uspi, zapote niño
Chrysobalanaceae	Hirtellaracemosavar.hexandra (Willd. ex Roem. & Schult.) Prance	bejuco limón, escobilla, tallepo
Chrysobalanaceae	Licania platypus (Hemsl.) Fritsch	cabeza de mono, caca de niño, huicume, menso zapote, zapote amarillo, zapote cabello, zapote de mono
Cleomaceae	*Cleomemagnifica* Briq.	chichiquelite
Clusiaceae	*Clusiaguatemalensis* Hemsl.	higo
Clusiaceae	*Garciniaintermedia* (Pittier) Hammel	guo-guo, limoncillo, naranjillo, wuowo
Combretaceae	*Terminalia amazonia* (J.F.Gmel.) Exell	canshán
Commelinaceae	*Tinantiaerecta* (Jacq.) Fenzl	hierba del pollo
Convolvulaceae	*Ipomoeabatatas* (L.) Lam.	camote, camote blanco, camote morado, coshlapa, mánta, quiebraplato
Convolvulaceae	*Ipomoeabracteata* Cav.	azalea de la barranca, bejuco blanco, camoteblanco, catispa, chile pato, papada de gallo
Convolvulaceae	*Ipomoeadumosa* (Benth.) L.O.Williams	campanilla, chonequi, corazón de la virgen, quiebraplato, xonequi
Cucurbitaceae	*Cucurbitaargyrosperma* Huber	calabaza, calabaza pipián, chigua, nipx (Totonaco)
Cucurbitaceae	*Cucurbitafoetidissima* Kunth	calabacilla loca
Cucurbitaceae	*Cucurbitamoschata* Duchesne	calabaza [fruit], calabaza criolla, pipián [seed]
Cucurbitaceae	*Cucurbitapepo* L.	ayoxochitl, ayoxochquilitl, calabacita, calabaza, puntas de calabaza [leaves], flor de calabaza [flowers], flor de pipiana, pipiana
Cucurbitaceae	*Cyclantheralangaei* Congn.	cincoquelites
Cucurbitaceae	*Melothriapendula* L.	pepinillo silvestre, sandía, sandía de ratón, sandía silvestre
Cucurbitaceae	*Sechiumedule* (Jacq.) Sw.	chayote, chayotli, erizo, güisquil, huisquil, jerizo, jurita, mayakg (Totonaco), usquil
Cyperaceae	*Schoenoplectustabernaemontani* (C.C.Gmel.) Palla	popoque, tule
Ebenaceae	Diospyrosacapulcensissubsp.verae-*crucis* (Standl.) Provance, I.García & A.C.Sanders	techona, zapotillo
Ebenaceae	*Diospyroscampechiana* Lundell	zapotito
Ebenaceae	*Diospyrosconzattii* Standl.	chapote, zapotillo
Ebenaceae	*Diospyrosnigra* (J.F.Gmel.) Perrier	zapote negro, zapote prieto, sawalh (Totonaco)
Ebenaceae	*Diospyrospalmeri* Eastw.	chapote
Ebenaceae	*Diospyrostexana* Scheele	chapote, chapote prieto
Ericaceae	*Arbutusxalapensis* Kunth	madroño, madrón
Ericaceae	*Comarostaphylisglaucescens* (Kunth) Zucc. ex Klotzsch	macuate
Ericaceae	*Comarostaphylispolifolia* (Kunth) Zucc. ex Klotzsch	macuate
Ericaceae	*Vacciniumleucanthum* Schltdl.	cahuiche, huicapol, huicapola, xoxocotzi
Euphorbiaceae	*Adeliabarbinervis* Cham. & Schltdl.	espinaca blanca
Euphorbiaceae	*Bernardiadodecandra* (Sessé ex Cav.) Govaerts	lisutkiwi
Euphorbiaceae	*Cnidoscolusaconitifolius* (Mill.) I.M.Johnst.	chaya
Euphorbiaceae	*Cnidoscolusmultilobus* (Pax) I.M.Johnst.	chaya, chaya amarilla, chaya brava, chaya mansa, chaya verde, mala mujer, pipian
Euphorbiaceae	*Cnidoscolustubulosus* (Miell.Sarg.) Johnst.	chaya, chaya amarilla, chaya brava, chaya mansa, chaya pica, chaya verde
Euphorbiaceae	*Crotonincanus* Kunth	salvia
Euphorbiaceae	*Crotonlindheimerianus* Scheele	salvia
Euphorbiaceae	*Crotonniveus* Jacq.	vara blanca
Euphorbiaceae	*Jatrophacurcas* L.	ashté, axté, chote, chút, pichoco, pipián, piñón, piñoncillo
Euphorbiaceae	*Jatrophadioica* Sessé	piñón
Euphorbiaceae	*Manihotpringlei* S.Wats.	yuca
Euphorbiaceae	*Manihotsubspicata* Rogers & Appan	papagallo
Fagaceae	Fagusgrandifoliasubsp.mexicana (Martínez) A.E.Murray	haya
Fagaceae	*Quercuscandicans* Née	almaizeoque, encino blanco, hoja china
Fagaceae	*Quercusemoryi* Torr.	encino prieto
Fagaceae	*Quercuspolymorpha* Schltdl. & Cham.	encino blanco
Fouquieriaceae	*Fouquieriasplendens* Engelm.	cardo santo
Gunneraceae	*Gunneramexicana* Brandegee	capa de pobre
Heliconiaceae	*Heliconialatispatha* Benth.	plantanillo
Heliconiaceae	*Heliconiaschiedeana* Klotzsch	costilla de ratón, papatla, papatlilla, plantanillo
Heliconiaceae	*Heliconiauxpanapensis* C.Gut.Báez.	plantanillo
Icacinaceae	*Oecopetalummexicanum* Greenm. & C.H.Thomps.	cacaté, cachichín
Iridaceae	*Tigridiapavonia* (L.f.) DC.	carcomeca
Juglandaceae	*Caryaillinoinensis* (Wangenh.) K.Koch	nogal, nogal criollo, nuez de cáscara de papel, nuez fina [fruits]
Juglandaceae	*Caryaovata* (Mill.) K.Koch	nogal, nogal americano
Juglandaceae	*Juglansmollis* Engelm.	nogal, nogal cimarrón
Juglandaceae	*Juglansolanchana* Standl. & L.O.Williams	cedro blanco, cedro nogal
Juglandaceae	*Juglanspyriformis* Liebm.	cedro nogal, nogal, nogal cimarrón
Lamiaceae	*Volkamerialigustrina* Jacq.	orégano
Lamiaceae	*Poliominthalongiflora* A.Gray	crespa
Lamiaceae	*Salviaballotiflora* Benth.	moste, musté
Lauraceae	*Beilschmiediaanay* (S.F.Blake) Kosterm.	aguacate de puerco, anay, anáy, anayo, escalán
Lauraceae	*Cinnamomumgrisebachii* Lorea-Hern.	canela
Lauraceae	*Litseaglaucescens* Kunth	laurel
Lauraceae	*Perseaamericana* Mill.	aguacate, aguacatillo, kunalhit, kuka'ta
Lauraceae	*Persealiebmannii* Mez.	agacate silvestre, sasafrás
Lauraceae	*Persealongipes* (Schltdl.) Meisn.	pahua
Lauraceae	*Perseaschiedeana* Nees	aguacatillo, chinin, chinín, chinine, lhpaw, pagua, pahua
Leguminosae	*Acaciellaacatlensis* Benth.	árbol de borrego, borreguitos, chindata, chivos, chondata, cornizuelo, cuernosuelo, guayalote, guayote, tlahuitole, yepaquilitl
Leguminosae	*Acaciellaangustissima* (Miller) Kuntze	framboyancillo
Leguminosae	*Acaciellacornigera* (L.) Willd.	cuerno de toro
Leguminosae	*Acaciellagreggii* Gray	gatuño
Leguminosae	*Acaciellasphaerocephala* Schlecht.	cornezuelo
Leguminosae	*Acaciellawrightii* Benth	uña de gato negra
Leguminosae	*Caesalpiniapulcherrima* (L.) Schwartz	tabachín
Leguminosae	*Canavaliaglabra* (M.Martens & Galeotti) J.D.Saue	flor de sacramento, pillo, sacalamente, sacramento, xokichay
Leguminosae	*Canavaliavillosa* Benth.	gallo
Leguminosae	*Cerciscanadensis* L.	duraznillo, palo de judas, pata de vaca
Leguminosae	*Crotalarialongirostrata* Hook. & Arn.	chipil, chipilan, chipile, chipilín, chipilino
Leguminosae	*Crotalariamaypurensis* HBK	chipilín
Leguminosae	*Dialiumguianense* (Aubl.) Sandwith	guach, guapaque, guapiqui, palo lacandón palo de lacandón, paque, paquí, tamarindo silvestre, uach
Leguminosae	*Diphysaamericana* (Mill.) M.Sousa	amarillo, camaroncillo, chipilcoi, chipilcoite, chipilín, coachepil, guachipilín, matansiyat, quebrancha, quebranche, quebrancho, quibrancha, rambai, tenquiques
Leguminosae	*Ebenopsisebano* (Bernand) Barneby & Grimes	ébano
Leguminosae	*Enterolobiumcyclocarpum* (Jacq.) Griseb	guanacastle, nacaste, nacastle, nacaxtle, parota
Leguminosae	*Erythrinaamericana* Mill.	alcaparra, chontal, colorín, equimexóchitl, equimite, gasparito, lhalhni, madre, madre del cacao, mote, pichoco, pito
Leguminosae	*Erythrinafolkersii* Krukoff & Moldenke	alcaparra, chiil, chocolín, chumpancle, colorín, equimite, espadita, flor de pita, flor de pitillo, flor de pito, gallitos, gasparita, gasparito, gásparo, lalhni, machetito, madre, patol, pemuche, pemuchi, permuche, pichocho, pichoco, pichojo, pispirique, poró, quemique, tlalhne, tsentse tsentse, tzonpantli, xompantli, zacapemucho
Leguminosae	*Erythrinalanata* Rose	colorín
Leguminosae	*Erythrinaherbacea* L.	alcaparra, chiil, chocolín, chumpancle, colorín, equimite, espadita, flor de pita, flor de pitillo, flor de pito, gallitos, gasparita, gasparito, gásparo, lalhni, machetito, madre, patol, pemuche, pemuchi, permuche, pichocho, pichoco, pichojo, pispirique, poró, quemique, tlalhne, tsentse tsentse, tzonpantli, xompantli, zacapemucho
Leguminosae	*Gliricidiasepium* (Jacq.) Walp.	cacahuananche, chuchunuc, cocohuite, cocomuite, cocuite, cocuitle, flor de San José, flor de sol, gallitos, mataratón, muiti, palo de sol, xab-yaab
Leguminosae	*Hymenaeacourbaril* L.	cuapinol, guapinol, guapinole
Leguminosae	*Ingaacrocephala* Steud.	vaina
Leguminosae	*Ingabrevipedicellata* Harms	ND
Leguminosae	*Ingajinicuil* Schltdl.	algodoncillo, chalahuite, chalahuitillo, cuajinicuil, jinicuil, kalam (Totonaco), xinicuil, vaina
Leguminosae	*Ingalaurina* (Sw.) Willd.	chelele
Leguminosae	*Ingapaterno* Harms.	aguatope, chalahuite, cuil machetón, jinicuil, jinicuil de vaina ancha, paterna, paterno, pepeto, vainillo
Leguminosae	*Ingapunctata* Willd.	chelele, taastk (Núntaha’yi), vaina
Leguminosae	*Ingasapindoides* Willd.	guatope
Leguminosae	*Ingasemialata* (Vell.) Mart.	ND
Leguminosae	*Ingavera* Willd.	vaina peluda
Leguminosae	*Ingasinacae* M.Sousa & Ibarra-Maríquez	chalahuite, cuajinicuil, guatope, jacanicuil, jinicuile
Leguminosae	*Leucaenaglauca* L.	lileac (Totonaco), liliaqui
Leguminosae	*Leucaenaleucocephala* (Lam.) de Wit	cola de zorro, guaje, guaje blanco, guajeverde, huaje, huashe, liliaque, slalak (Totonaco)
Leguminosae	*Leucaenapulverulenta* (Schltdl.) Benth.	guajillo, tepehuaje
Leguminosae	*Macroptiliumgibbosifolium* (Ortega) A.Delgado	frijol chichimeque
Leguminosae	*Pachyrhizuserosus* L.Urb	jícama
Leguminosae	*Phaseoluscoccineus* L.	ayacote, chichimeque, frijol boti, frijol burro, frijol de monte, frijol gordo, chachan, chachana, flor de bótil, flor de frijol, flor de quelite, hachana, mahtlaketl, xaxan, xaxana, xochikilitl, xochimaríah
Leguminosae	*Phaseolusvulgaris* L.	ashlañ bu’ul, ejote, frijol negro, sic (Núntaha’yi), stapu (Totonaco)
Leguminosae	*Pithecellobiumdulce* (Roxb.) Benth	cuamuchil, guamoche, guamuche, guamúchil, guaymochile, huamuchil, humo, nempa, pinzán, tucuy, umon
Leguminosae	*Pithecellobiumlanceolatum* (Humb. & Bonpl. ex Willd.) Benth	guamúchil ahogador, peleple
Leguminosae	*Pithecellobiumpachypus* Pittier	guamúchil
Leguminosae	*Prosopisglandulosa* Torr.	mezquite
Leguminosae	*Prosopisjuliflora* (Sw.) DC.	mezquite
Leguminosae	*Prosopislaevigata* (Willd.) M.C.Johnston	mezquite de árbol
Leguminosae	*Prosopistamaulipana* Burkart.	mezquite
Leguminosae	*Sennafruticosa* (Mill.) H.S.Irwin & Barneby	quelite
Leguminosae	*Sennapapillosa* (Britton & Rose) H.S.Irwin & Barneby	quelite
Loasaceae	*Eucnidebartonioides* Zucc.	lechuguilla
Magnoliaceae	*Magnoliamexicana* DC.	aguacote, árbol de corazón, flor de atole, flor de corazón, flor de rosa, kuwi xa’nat, magnolia, moniakuy, moñaykuy-imayak, moynacoy, sochilmoynacoy, súchil, xolochochitl, yolo, yololxochitl, yoloshanat, yolos chil, yolosúchil, yoloxochitl
Magnoliaceae	*Magnoliazoquepopolucae* A.Vázquez	súchil (Náhuatl), moiñacoi-imayak, moñacoi-imaya, moñacoy, monaikoi-imayak, moniacuy, moñiacuy, mooyniak-cuy, mou-ña-coy, moyñacoi, moynacoy, all meaning “tree with flower coming from an eggshell” (Zoque-popoluca)
Malpighiaceae	*Bunchosialindeniana* A.Juss.	árbol manchado, zapotillo
Malpighiaceae	*Byrsonimacrassifolia* (L.) Kunth	nananche, nance, nanche, na chi (Núntaha’yi), nanche, nanche agrio, nance, peraleja
Malpighiaceae	*Galphimiaglauca* (Cav.) Kuntze	cola de zorro
Malpighiaceae	*Malpighiaglabra* L.	camaroncito, cereza, ciruela cimarrona, grosella roja, lumbre, nance, manzanita, panecito, palo de tomatillo
Malpighiaceae	*Mascagniamacroptera* (Moc. & Sessé ex DC.) Nied	gallinita
Malvaceae	*Anodacristata* (L.) Schldl.	malvavisco
Malvaceae	*Ceibaaesculifolia* (Kunth) Britton & Baker f.	ceiba, pochota, pochote
Malvaceae	*Ceibapentandra* (L.) Gaertn.	ceiba
Malvaceae	*Guazumaulmifolia* Lam.	acashti, ajillá, aquiche, guácima, guácimo, guazima, guázumo
Malvaceae	*Hibiscusmartianus* Zucc.	manzanita
Malvaceae	*Malvaviscusarboreus* Cav.	bejuquillo, catapachat (Totonaco), chanita, chocho, chinina, civil monacillo, farolito, fekom, flor de santos, gagapache, ixwaquelt, ixwaquen, majaguilla, manzanilla, manzanillo, manzanita, mazapan, monacillo rojo, monacillo, obelisco de la sierra, panelita, paniqueso, plumagillo, teresita, totopotzin, totopatzín, totopoxin, tulipán, tulipancito del monte, sibil, xtut-quene
Malvaceae	*Malvaviscusoaxacanus* Standl.	chocho, tulipancillo
Malvaceae	*Pachiraaquatica* Aubl.	acamoyote, apompo, axilochóchitl, chanacol blanco, clavellina blanca, zapote de agua, zapote bobo, zapote reventador, zapotón
Malvaceae	*Pseudobombaxellipticum* (Kunth) Dugand	flor de mota, juanjilón
Malvaceae	*Quararibeafunebris* (La Llave) Vischer	árbol canelo, árbol de funeral, árbol de molinillo, cacahuaxochitl, cacaoxochitl, canela, canelita, flor de cacao, huacanelo, kiwi pobostatli, madre de cacao, maricacao, molinillo, palo de canela, palo de chocolate, palo volador, rosa de cacao, rosita de cacao
Malvaceae	Quararibeayunckerisubsp.sessiliflora Standl.	ND
Malvaceae	*Sterculiaapetala* (Jacq.) H.Karst.	castañas, castaño, pepetaca
Malvaceae	*Sterculiamexicana* R.Br	bellota
Marantaceae	*Calathealutea* (Aubl.) E.Mey. ex Schult.	berijo, berijado, berijao, hoja de berijado, hojablanca, hoja de té, hoja de verijado, tho
Marantaceae	*Calatheamacrosepala* K.Schum. (Aubl.) Lindl.	chochogo, chogo, chogogo, sauco, shuco, suco, xuco
Marantaceae	*Calatheamarantifolia* Standl.	chochogo, chonegue, choschogo, lechuga, shuco, suco, xoxogo, xuco
Marantaceae	*Goeppertiamisantlensis* (Lascurain) Borchs. & S.Suárez	hoja redonda, papelillo
Marantaceae	*Goeppertiaovandensis* (Matuda) Borchs. & S.Suárez	plantanillo
Marantaceae	*Marantaarundinacea* L.	apichillo, azafrán, chancla, hierba martina, hua-ja (Popoluca), papatilla, platanillo, t'aau' (Huasteco), sagú, yuquilla
Marantaceae	*Stromanthemacrochlamys* (Woodson & Standl.) H.A.Kenn. & Nicolson	hoja de piedra, malintzi, tompimil, tonpimil
Martyniaceae	*Martyniaannua* L.	ND
Melastomataceae	*Conostegiaicosandra* (Sw. ex Wikstr.) Urb.	ojo de venado
Melastomataceae	*Conostegiaxalapensis* (Bonpl.) D.Don ex DC.	capulín, capulín de cotorro, chicab, cinco negritos, hojalatillo blanco, sedita, serita, tecapulín, teshuate, tezhualillo
Melastomataceae	*Heterocentronsubtriplinervium* (Link & Otto) A.Braun & C.D.Bouché	caña de león
Menispermaceae	*Cocculuscarolinus* (L.) DC.	hierba de ojo
Moraceae	*Brosimumalicastrum* Sw.	apomo, nazareno, ocoxiltli, ojite, ojoche, osh, ramón, samaritano
Moraceae	*Ficusaurea* Nutt.	higo
Moraceae	*Ficuscotinifolia* Kunth	higuerón
Moraceae	*Ficuspertusa* L.f.	amate
Moraceae	*Macluratinctoria* (L.) D.Don ex Steud.	chichiti, mora, moradilla, moral, moral amarillo, palo amarillo
Moraceae	*Poulseniaarmata* (Miq.) Standl.	abasbabi, ababábite, agabasgabi, aguatoso, carnepescado, carne de pescado, carnero, carnero blanco, chagane, chirimoya, huichilama, masamorro
Moraceae	*Pseudolmediaglabrata* (Liebm.) C.C.Berg	ojoche colorado, ramón colorado, ramón de mico, tepetomate, tomatillo
Moraceae	*Pseudolmediaoxyphyllaria* Donn.Sm.	manash, tepetomate, tomatillo, wáxax (Totonaco)
Moraceae	*Trophisracemosa* (L.) Urb.	campanilla, ramón, ramón colorado, ramoncillo, papelillo
Muntingiaceae	*Muntingiacalabura* L.	capulín, capulín real, capulincillo, cerezo, nigua, nigüito, poan, puam, puan, puan capulín, puyam (Totonaco)
Myrtaceae	*Calyptranthesschiedeana* O.Berg.	guayabillo
Myrtaceae	*Eugeniaacapulcensis* Steud.	capulín
Myrtaceae	*Eugeniacapuli* (Schltdl. & Cham) Hook. & Arn.	capulín, capulín agarroso, capulín de zorrillo, frutilla, escobilla, escobillo, guayabillo cimarrón, palo de temazate, pimientilla
Myrtaceae	*Eugeniaoerstediana* O.Berg	capulín guinda, escobilla, rainjan
Myrtaceae	*Eugeniaxalapensis* Kunth	gallito
Myrtaceae	*Myrciariafloribunda* (H.West ex Willd.) O.Berg	arrayán
Myrtaceae	*Mosieraehrenbergii* (O.Berg) Landrum	chepucuy, escobilla
Myrtaceae	*Pimentadioica* (L.) Merr.	pimienta, pimenta gorda, u'cun (Totonaco), uc-suc (Popoluca), xocoxo'chitl (Nahuatl)
Myrtaceae	*Psidiumfriedrichsthalianum* (O.Berg) Niedenzu	guayaba cimarrona, guayaba agria, guayabo agrio, guayabo de monte
Myrtaceae	*Psidiumsartorianum* (O.Berg) Nied.	arrayán, 'cales'ni (Misanteco), capulín, guayaba de tejón, guayabillo
Nelumbonaceae	*Nelumbolutea* (Willd.) Pers.	ayacastle, flor de agua amarilla, malacate, pulul
Olacaceae	*Ximeniaamericana* L.	ciruelillo
Oleaceae	*Forestieraangustifolia* Torrey	panalero
Orchidaceae	*Vanillaplanifolia* Jacks. ex Andrews	vainilla
Orchidaceae	*Vanillapompona* Schiede	vainilla pompona
Orobanchaceae	*Escobedialaevis* Schltdl. & Cham.	azafran de raíz, azafrancillo
Oxalidaceae	*Oxalislatifolia* Kunth.	agrito
Passifloraceae	*Passifloraambigua* Hemsl.	gagapache, jujo
Passifloraceae	*Passifloracookii* Killip	ND
Passifloraceae	*Passifloramexicana* Juss.	ND
Passifloraceae	*Passifloraserratifolia* L.	granada del monte, pasión
Passifloraceae	*Turneradiffusa* Wilid.	damiana, hierba del venado, venadita
Phytolaccaceae	*Phytolaccaamericana* L.	jabonera
Phytolaccaceae	*Phytolaccaicosandra* L.	cóngora, jabonera, jabonero, jorja, tonga
Phytolaccaceae	*Phytolaccarivinoides* Kunth & Bouché	guanchaparrón, jaboncillo, jorga, quelite rojo, joklhkgk (Totonaco)
Picramniaceae	*Picramniateapensis* Tul.	muste, mutza
Pinaceae	*Pinuscembroides* Zucc.	pino piñonero [plant], piñón [seed]
Pinaceae	*Pinusnelsonii* Shaw	pino piñonero [plant], piñón duro [seed]
Pinaceae	*Pinuspinceana* Gordon	pino piñonero
Piperaceae	*Peperomiaasarifolia* Schltdl.	ND
Piperaceae	*Peperomiaberlandieri* Miq.	ND
Piperaceae	*Peperomiahobbitoides* T.Wendt	ND
Piperaceae	*Peperomiamaculosa* (L.) Hook.	cilantro de monte, cilantro macho, najashuio macho
Piperaceae	*Peperomiapeltilimba* C.DC. ex Trel.	cilantro cimarrón, cilantro de monte, nacazgüillo, limonascagüillo, oreja de burro, tequelite
Piperaceae	*Peperomiarotundifolia* (L.) Kunth	caminante
Piperaceae	*Piperamalago* L.	cordoncillo
Piperaceae	*Piperauritum* Kunth	acoyo, acuyo, aguiyu, alahan, cordón blanco, cordoncillo, hierba de santa maría, hierba santa, hoja santa, homequelite mecaxóchitl, momo, mumu, omequelite, pimienta sagrada, tlanepa quelite, tlanepa, tlanepaquilitl, totzoay
Poaceae	*Zeamays* L.	cabellos de elote, elote, jilote, maíz, milpa, totomoxtle [dry leaves], hojas de maíz [freshleaves], moc (Núntaha’yi), Kúxi (Totonaco)
Podocarpaceae	*Podocarpusmatudae* Lundell	palmilla
Polygonaceae	*Coccolobabarbadensis* Jacq.	tepelcahuite, uvero
Polygonaceae	*Coccolobauvifera* L.	carnero, uva de playa, uvero de mar
Primulaceae	*Ardisiacompressa* Kunth	capulín de mayo, capulín de tejón, capulín silvestre, capulincillo, chagalapoli, chico correoso
Primulaceae	*Ardisiaescallonioides* Schltdl. & Cham.	capulín, matan'kiwi (Totonaco)
Primulaceae	*Ardisianigropunctata* Oerst.	capulín
Primulaceae	*Parathesispsychotrioides* Lundell	capulín, chagalapoli, silling, akgtalawat (Totonaco)
Primulaceae	*Parathesisserrulata* (Sw.) Mez	capulín arribeño, capulín de sabana
Rhamnaceae	*Ceanothuscoeruleus* Lagasca & Gen	palo colorado
Rhamnaceae	*Ceanothusgreggii* A.Gray	palo dezorrillo
Rhamnaceae	*Colubrinaelliptica* (Sw.) Brizicky & Stern	guayacán, manzanita
Rhamnaceae	*Colubrinagreggii* S.Watson	manzanilla
Rhamnaceae	*Condaliahookeri* M.C.Johnst.	brasil
Rhamnaceae	*Condalialycioides* (A.Gray) Weberb	crucillo
Rhamnaceae	*Karwinskiamollis* Schltdl.	tullidor
Rhamnaceae	*Rhamnushumboldtiana* Willd. ex Schult.	coyotillo, tullidor
Rhamnaceae	*Ziziphusamole* (Sessé & Moc.) M.C.Johnst.	naranjillo
Rosaceae	*Crataegusmexicana* Moc. & Sessé ex DC.	cainúm, chisté, tejocote, manzanilla, manzanillo
Rosaceae	*Crataegusrosei* Eggl.	tejocote, tejocote cimarrón
Rosaceae	*Fragariavesca* L.	fresa, fresa cimarrón
Rosaceae	*Prunusserotina* Ehrh.	capulín
Rosaceae	*Rubusadenotrichus* Schltdl.	mora, morash, zarza, zarzamora
Rosaceae	*Rubusapogaeus* L.H.Bailey	zarzamora
Rosaceae	*Rubuscoriifolius* Liebm	zarzamora silvestre
Rosaceae	*Rubushumistratus* Steudel	zarzamora
Rosaceae	*Rubussapidus* Schltdl.	zarzamora, zarzamora silvestre
Rosaceae	*Rubustrivialis* Michx.	zarzamora
Rubiaceae	*Alibertiaedulis* (L. Rich.) A.Rich. ex DC.	castarrica blanca
Rubiaceae	*Chiococcaalba* (L.) Hitch	perlilla
Rubiaceae	*Genipaamericana* L.	jagua, jagua blanca, jagüe, jahue, maluco, yual, yoale, xagua
Rubiaceae	*Hameliapatens* Jacq.	hierba de la mula
Rubiaceae	*Mitchellarepens* L.	mora de codorniz
Rutaceae	*Casimiroaedulis La Llave* & Lex.	pera mexicana, zapotillo, zapote blanco
Rutaceae	*Casimiroagreggii* (S.Watson) F.Chiang	chapote amarillo
Rutaceae	*Casimiroapringlei* (S.Watson) Engl.	limoncillo
Rutaceae	*Pteleatrifoliata* L.	pinacatillo
Rutaceae	*Zanthoxylumfagara* (L.) Sarg.	colima
Salicaceae	*Xylosmaflexuosa* (Kunth) Hemsl.	capulín corona, coronilla, palo de brujo
Sapindaceae	*Paulliniatomentosa* Jacq.	arete de novia
Sapotaceae	*Chrysophyllummexicanum* Brandegee ex Standl.	caimito, caimito cimarrón, caimito del monte, caimito silvestre, caimitillo, pistillo
Sapotaceae	*Chrysophyllumvenezuelanense* (Pierre) T.D.Penn.	chicozapote del monte
Sapotaceae	*Manilkarachicle* (Pittier) Hammel	chicozapote, zapote chicle, zapote chico, sapotilla, shenc, sculu-jaca (Totonaco)
Sapotaceae	*Pouteriacampechiana* (Kunth) Baehni	caca de niño, cucumú, kanistú, kukun (Totonaco), mante, nochi, tapa, zapote agrio, zapote amarillo, zapote mante, zapote niño
Sapotaceae	*Pouteriaglomerata* (Miq.) Radlk.	chocho
Sapotaceae	*Pouteriasapota* (Jacq.) H.E.Moore & Stearn	atzapotlcuahuitl (Náhuatl), cuyg’ auac (Popoluca), lankájaka (Totonaco), mamey, mamey colorado, zapote, zapote mamey
Sapotaceae	*Sideroxyloncelastrinum* (Kunth) T.D.Penn.	coma cimarrona, coma resinera
Sapotaceae	*Sideroxylonpalmeri* (Rose) T.D.Penn.	coma real
Sapotaceae	*Sideroxylonpersimile* (Hemsl.) T.D.Penn.	ND
Sapotaceae	*Sideroxylonsalicifolium* (L.) Lam.	sapotillo
Scrophulariaceae	*Buddlejamarrubiifolia* Benth.	azafrán
Simaroubaceae	*Simaroubaglauca* DC.	aceituno negrito, gusano, pasaque, rabo de lagarto blanco, zapatero
Smilacaceae	*Smilaxaristolochiifolia* Mill.	zarzaparilla
Smilacaceae	*Smilaxbona*-*nox* L.	zarza, zarzaparilla
Smilacaceae	*Smilaxdomingensis* Willd.	asquiote, axquiote, azquiote, bejuco de canasta, bejuco de membrillo, bejuco de zarzaparrilla, chiquihuite, cocolmeca, cocolmecate, colcomeca, corcomeca, uut’ ts’aah, zarzaparrilla, bigote de acamayo, kgansalis
Smilacaceae	*Smilaxmollis* Willd.	zarzaparilla
Smilacaceae	*Smilaxmoranensis* M.Martens & Galeotti	zarzaparilla
Smilacaceae	*Smilaxspinosa* Mill.	zarzaparilla
Solanaceae	*Capsicumannuum* L.	amashito, chile de árbol, chiles de color, chile pico paloma, chile piquín, dulce, jalapeño, pimiento morrón, poblano, serrano, tsílampin (Totonaco)
Solanaceae	*Cestrumracemosum* Ruiz & Pav.	amargoso
Solanaceae	*Jaltomataprocumbens* (Cav.) J.L.Gentry	acahualera, chaltotongo, cojudo, equelite, jaltomata, quelite tomaquelite, tomatillo, tompis
Solanaceae	*Lyciumberlandieri* Dunal	cilindrillo
Solanaceae	*Lyciumcarolinianum* Walt	saladilla
Solanaceae	*Margaranthussolanaceus* Schltdl.	ND
Solanaceae	*Physalisangulata* L.	tomatillo
Solanaceae	*Physalisarborescens* L.	tomatillo de monte
Solanaceae	*Physaliscinerascens* (Dunal) Hitch.	tomatillo
Solanaceae	*Physalisgracilis* Miers	champ lulh (Totonaco), costomate, tomate, tomatillo
Solanaceae	*Physalisixocarpa* Brot. ex Hornem.	tomate de cáscara, túmat (Totonaco)
Solanaceae	*Physalislobata* Torrey	tomatillo
Solanaceae	*Physalismelanocystis* (B.L.Rob.) Bitter	tomate verde
Solanaceae	*Physalisorizabae* Dunal	juatomate amarillo, tomate de bota
Solanaceae	*Physalisphiladelphica* Lam.	tomate, tomate verde, tomate de cascara, tomatillo, tomatillo de labor
Solanaceae	*Physalispringlei* Greenman	tomatillo
Solanaceae	*Physalispubescens* L.	miltomate, tomate, tomatillo
Solanaceae	*Physalisviscosa* Gray	ND
Solanaceae	*Solandraguttata* D.Don	ND
Solanaceae	*Solandramaxima* (Sessé & Moc.) P.S.Green	copa de oro, pera tetona, tetona
Solanaceae	*Solanumamericanum* Mill.	hierbamora, hierba mora, mustulut (Totonaco)
Solanaceae	*Solanumappendiculatum* Dunal	tinguarache
Solanaceae	*Solanumlycopersicum* L.	jitomate, tomate citlali, tomate coyote, tomate riñon, tomate silvestre
Solanaceae	*Solanumnigrescens* M.Martens & Galeotti	jitomate, tomate rojo
Solanaceae	*Solanumcandidum* Lindley	hierba mora, quelite morado, yerbamora
Solanaceae	*Solanumoxycarpum* Schiede	papa cimarrona
Solanaceae	*Solanumverrucosum* Schldl.	ND
Solanaceae	*Witheringiameiantha* (Donn.Sm.) Hunz.	cuña
Talinaceae	*Talinumfruticosum* (L.) Juss.	verdolaga
Typhaceae	*Typhadomingensis* Persoon	tule
Ulmaceae	*Ampelocerahottlei* (Standl.) Standl.	guaya de monte, wayam
Ulmaceae	*Phyllostylonrhamnoides* (J.Poiss.) Taub.	cerón
Verbenaceae	*Citharexylumberlandieri* Robb.	negrito, orejuela, revientacabra
Verbenaceae	*Citharexylumbrachyanthum* A.Gray	agrito
Verbenaceae	*Lippiaalba* (Miller) N.E.Br.	orégano
Verbenaceae	*Lippiagraveolens* Kunth	hierba dulce, orégano, orégano hoja chica, oreganillo, salve real
Vitaceae	*Vitiscinerea* (Engelm) Millardet	uva de monte
Vitaceae	*Vitismustangensis* Buckley	uva de monte
Vitaceae	*Vitistiliifolia* Humb. & Bonpl. ex Schult.	bejuco de agua, bejuco de parra, parra broncadora, parra silvestre, tecamate, tripas de vaca, sánalo todo, uva, uva de monte, uvilla cimarrona
Zamiaceae	*Dioonedule* Lindley	chamal, palma de teresita, quiotamal, tiotamal
Zamiaceae	*Dioonspinulosum* Dyer ex Eichl.	chicalito [seeds], palma de chicalite [plant]
Zingiberaceae	*Renealmiaalpinia* (Rottb.) Maas	guá
Zingiberaceae	*Renealmiamexicana* Klotzsch ex Petersen	bex, tapicón, hoja de bexo
Zygophyllaceae	*Neoschroeteratridentata* (Sessé & Moc. ex DC.) Briq	gobernadora

## References

[B7591415] Aguilar Vásquez Yunin, Caso Barrera Laura, Aliphat Fernández Mario (2019). Agroecosistemas tradicionales Núntaha’yi en la Reserva de la Biósfera Los Tuxtlas, Veracruz, México. Región y Sociedad.

[B7591464] APG IV (2016). An update of the Angiosperm Phylogeny Group classification for the orders and families of flowering plants: APG IV. Botanical Journal of the Linnean Society.

[B7591070] México Atlas de los Pueblos Indigenas de Instituto Nacional de los Pueblos Indigenas/Instituto Nacional de las Lenguas Indigenas. http://atlas.cdi.gob.mx.

[B7591424] Avilez-López Teresita, van der Wal Hans, Aldasoro-Maya Elda Miriam, Rodríguez-Robles Ulises (2020). Home gardens’ agrobiodiversity and owners’ knowledge of their ecological, economic and socio-cultural multifunctionality: A case study in the lowlands of Tabasco, México. Journal of Ethnobiology and Ethnomedicine.

[B7573634] Bacchetta Loretta, Visioli Francesco, Cappelli Giulia, Caruso Emily, Martin Gary, Nemeth Eva, Bacchetta Gianni, Bedini Gianni, Wezel Alexander, van Asseldonk Tedje, van Raamsdonk Leo, Mariani Francesca, on behalf of the Eatwild Consortium (2016). A manifesto for the valorization of wild edible plants. Journal of Ethnopharmacology.

[B7573726] Bhatia Harpreet, Sharma Yash Pal, Manhas R. K., Kumar Kewal (2018). Traditionally used wild edible plants of District Udhampur, J&K, India. Journal of Ethnobiology and Ethnomedicine.

[B7591473] Borsch Thomas, Berendsohn Walter, Dalcin Eduardo, Delmas Maïté, Demissew Sebsebe, Elliott Alan, Fritsch Peter, Fuchs Anne, Geltman Dmitry, Güner Adil, Haevermans Thomas, Knapp Sandra, Roux M. Marianne, Loizeau Pierre‐André, Miller Chuck, Miller James, Miller Joseph T., Palese Raoul, Paton Alan, Parnell John, Pendry Colin, Qin Hai‐Ning, Sosa Victoria, Sosef Marc, Raab‐Straube Eckhard, Ranwashe Fhatani, Raz Lauren, Salimov Rashad, Smets Erik, Thiers Barbara, Thomas Wayt, Tulig Melissa, Ulate William, Ung Visotheary, Watson Mark, Jackson Peter Wyse, Zamora Nelson (2020). World Flora Online: Placing taxonomists at the heart of a definitive and comprehensive global resource on the world's plants. Taxon.

[B7591433] Bye R, Linares E (1983). The role of plants found in the Mexican markets and their importance in ethnobotanical studies. Journal of Ethnobiology.

[B7591442] Bye R, Ramamoorthy TP, Bye R, Lot A, Fa J (1993). Biological Diversity of Mexico: Origins and Distribution.

[B7591455] Caballero Javier, Casas Alejandro, Cortés Laura, Mapes Cristina (1998). Patrones en el conocimiento, uso y manejo de plantas en pueblos indígenas de México. Estudios Atacameños. Arqueología y Antropología Aurandinas..

[B7591201] Cáceres Armando, Cruz Sully M. (2019). Edible seeds, leaves and flowers as Maya super foods: function and composition. International Journal of Phytocosmetics and Natural Ingredients.

[B7591220] Casas Alejandro, Vázquez María del Carmen, Viveros Juan Luis, Caballero Javier (1996). Plant management among the Nahua and the Mixtec in the Balsas River Basin, Mexico: an ethnobotanical approach to the study of plant domestication. Human Ecology.

[B7591178] Centurión-Hidalgo Dora, Espinosa-Moreno Judith, Cruz-Lázaro Efrain De la, Báez-Mendoza Lourdes, Sánchez-Ruiz Blanca Alicia, Pérez-Robles Leonor del Carmen (2019). Estacionalidad de los vegetales comercializados en los mercados públicos del estado de Tabasco. Estudios Sociales. Revista de Alimentación Contemporánea y Desarrollo Regional.

[B7573652] CEPAL (2018). Segundo informe anual sobre el progreso y los desafíos regionales de la Agenda 2030 para el Desarrollo Sostenible en América Latina y el Caribe. http://hdl.handle.net/11362/43415.

[B7591377] Chablé-Pascual R., Paloma-López DJ, Vázquez-Navarrete CJ, Ruíz-Rosado O, Mariaca-Méndez R., Ascencio-Rivera JM (2015). Estructura, diversidad y uso de las especies en huertos familiares de la Chontalpa, Tabasco, México. Ecosistemas y Recursos Agropecuarios.

[B7591306] Challenger A., Soberón J., Sarukhán J. (2008). Capital Natural de México, vol. I: Conocimiento Actual de la Biodiversidad.

[B7573625] Chivenge Pauline, Mabhaudhi Tafadzwanashe, Modi Albert, Mafongoya Paramu (2015). The potential role of neglected and underutilised crop species as future crops under water scarce conditions in Sub-Saharan Africa. International Journal of Environmental Research and Public Health.

[B7717207] Christenhusz Maarten J. M., Byng James W. (2016). The number of known plants species in the world and its annual increase. Phytotaxa.

[B7573612] Collins W., Hawtin GC, Collins Wanda W., Qualset Calvin O., CO (1999). Biodiversity in Agroecosystems.

[B7591327] Córdoba y Ordóñez Juan (2004). La agricultura en México: un atlas en blanco y negro. Investigaciones Geográficas.

[B7573594] Corlett Richard T. (2016). Plant diversity in a changing world: status, trends, and conservation needs. Plant Diversity.

[B7591388] Cruz MC, López H de Luna (2018). El aprovechamiento de los productos de la naturaleza por los indígenas de Chicontepec, Veracruz. Contextualizaciones Latinoamericanas.

[B7591097] Del Angel-Pérez Ana Lid, Mendoza MA (2004). Totonac homegardens and natural resources in Veracruz, Mexico. Agriculture and Human Values.

[B7573660] Dop Marie Claude, Kefi Fayçal, Karous Olfa, Verger Eric O, Bahrini Asma, Ghrabi Zeineb, El Ati Jalila, Kennedy Gina, Termote Céline (2020). Identification and frequency of consumption of wild edible plants over a year in Central Tunisia: a mixed-methods approach. Public Health Nutrition.

[B7573675] FAO (2019). Informe anual 2018 America Latina y el Caribe. http://www.fao.org/3/ca4222es/ca4222es.pdf.

[B7591210] Fedick Scott L., De Lourdes Flores Delgadillo Maria, Sedov Sergey, Rebolledo Elizabeth Solleiro, Mayorga Sergio Palacios (2008). Adaptation of Maya homegardens by “container gardening” in limestone bedrock cavities. Journal of Ethnobiology.

[B7573585] Garn Stanley M., Leonard William R. (1989). What did our ancestors eat?. Nutrition Reviews.

[B7591160] GBIF.org GBIF: The Global Biodiversity Information Facility. https://www.gbif.org.

[B7591229] Gutiérrez-­Santillán Tania Vianney, Moreno­-Fuentes Ángel, Sánchez­-González Arturo, Sánchez­-Rojas Gerardo (2019). Knowledge and use of biocultural diversity by Nahua in the Huasteca region of Hidalgo, Mexico. Ethnobiology and Conservation.

[B7591142] Hernández Luis, Romo Claudia González, Medrano Francisco González (1991). Plantas útiles de Tamaulipas. Anales del Instituto de Biología. Serie Botánica. UNAM.

[B7652423] Hijmans R. J., Phillips S., Leathwick J., Elith J. (2017). Dismo: Species distribution modeling.. https://CRAN.R-project.org/package=dismo..

[B7590930] Iturriaga José N. (2018). Biodiversidad y diversidad cultural de México: una cocina históricamente nutritiva y sostenible. U. México, el origen y la evolución de la producción de alimentos y su impacto en los patrones de consumo.

[B7573717] Jacob Michelle Cristine Medeiros, Araújo de Medeiros Maria Fernanda, Albuquerque Ulysses Paulino (2020). Biodiverse food plants in the semiarid region of Brazil have unknown potential: a systematic review. PLOS One.

[B7591297] Jáuregui E., Soto C. (1975). La vertiente del Golfo de México. Algunos aspectos fisiográficos y climáticos. Investigaciones Geográficas.

[B7573683] Kang Yongxiang, Łuczaj Łukasz, Kang Jin, Zhang Shijiao (2013). Wild food plants and wild edible fungi in two valleys of the Qinling mountains (Shaanxi, central China). Journal of Ethnobiology and Ethnomedicine.

[B7591126] Lascurain Maite, Avendaño Sergio, Amo Silvia del, Niembro Aníbal (2010). Guía de frutos silvestres comestibles en Veracruz.

[B7591406] López-Santiago AA, López-Santiago MA, Cunil-Flores JM, Medina-Cuéllar SE (2019). Valor socioeconómico de las plantas para una comunidad indígena Totonaca. Interciencia.

[B7573793] Mapes Cristina, Basurto Francisco, Lira Rafael, Casas Alejandro, Blancas José (2016). Ethnobotany of Mexico.

[B7591349] Mendoza-González G., Martínez M. L., Lithgow D., Pérez-Maqueo O., Simonin P. (2012). Land use change and its effects on the value of ecosystem services along the coast of the Gulf of Mexico. Ecological Economics.

[B7591257] Morrone JUAN J. (2005). Hacia una síntesis biogeográfica de México. Revista Mexicana de Biodiversidad.

[B7591266] Morrone Juan J. (2010). Fundamental biogeographic patterns across the Mexican transition zone: An evolutionary approach. Ecography.

[B7591238] Narváez-Elizondo R. E., González-Elizondo M., González-Elizondo M. S., Tena-Flores J. A., Castro-Castro A. (2020). Edible ethnoflora of the southern Tepehuans of Durango, México. Polibotánica.

[B7573709] Ozturk M., Hakeem K. R., Ashraf M., Ahmad M. S.A. (2018). Global perspectives on underutilized crops.

[B7591248] Ramírez García Adán Guillermo, Montes Renteria Rodolfo, Ramírez Miranda Cesar Adrian, Rodríguez Sauceda Elvia Nereyda (2020). Plantas con valor de uso para la etnia Yaqui en Sonora, México. Ra Ximhai.

[B7652432] Team R Core (2020). R: A Language and Environment for Statistical Computing. https://www.r-project.org.

[B7591397] Rooduijn Bastiaan, Bongers Frans, van der Wal Hans (2018). Wild native trees in tropical homegardens of southeast Mexico: fostered by fragmentation, mediated by management. Agriculture, Ecosystems & Environment.

[B7728179] Roullier Caroline, Duputié Anne, Wennekes Paul, Benoit Laure, Fernández Bringas Víctor Manuel, Rossel Genoveva, Tay David, McKey Doyle, Lebot Vincent (2013). Disentangling the Origins of Cultivated Sweet Potato (Ipomoeabatatas (L.) Lam.). PLOS One.

[B7591115] Ruíz-Carrera V., Peña-López EG, Lau-Vázquez SC, Maldonado-Mares F, Ascencio-Rivera JM, Guadarrama-Olivera MA (2004). Macronutrimentos de fitorrecursoso alimenticios de especies aprovechadas por grupos étnicos en Tabasco, México. Universidad y Ciencia.

[B7573701] Sánchez-Mata M. de Cortes, Tardío J. (2016). Mediterranean wild edible plants: ethnobotany and food composition tables.

[B7591134] Sánchez-Trinidad Lesterloon (2017). Las flores en la cocina Veracruzana en cocina indígena y popular 75 Ed. Secretaría de Cultura.

[B7573774] Saruhkán José, Halfter Gonzalo, Koleff Patricia, González R., Carabias Julia, March I. (2009). Capital natural de México. Síntesis: conocimiento actual, evaluación y perspectivas de sustentabilidad.

[B7590961] Silva Evodia, Lascurain Maite, Legarreta Alberto Peralta de (2016). Cocina y biodiversidad en México. CONABIO. Biodiversitas.

[B7591275] Soto-Esparza M., Giddings-Berger LE, Cruz-Angón (2011). La biodiversidad de Veracruz: estudio de estado l..

[B7591368] Soto M., Gama L., Gómez M. (2001). Los climas cálidos subhúmedos del estado de Veracruz, México. Foresta Veracruzana.

[B7591359] Steinberg Michael, Taylor Matthew, Kinney Kealohanuiopuna (2014). The El Cielo Biosphere Reserve: forest cover changes and conservation attitudes in an important neotropical region. The Professional Geographer.

[B7591336] Travieso-Bello A., Gómez R., Moreno-Casasola P., Moreno-Casasola P. (2006). Entornos Veracruzanos: la costa de la Mancha.

[B7573735] Ulian Tiziana, Diazgranados Mauricio, Pironon Samuel, Padulosi Stefano, Liu Udayangani, Davies Lee, Howes Melanie‐Jayne R., Borrell James S., Ondo Ian, Pérez‐Escobar Oscar A., Sharrock Suzanne, Ryan Philippa, Hunter Danny, Lee Mark A., Barstow Charles, Łuczaj Łukasz, Pieroni Andrea, Cámara‐Leret Rodrigo, Noorani Arshiya, Mba Chikelu, Nono Womdim Rémi, Muminjanov Hafiz, Antonelli Alexandre, Pritchard Hugh W., Mattana Efisio (2020). Unlocking plant resources to support food security and promote sustainable agriculture. Plants, People, Planet.

